# Coral larvae for restoration and research: a large-scale method for rearing *Acropora millepora* larvae, inducing settlement, and establishing symbiosis

**DOI:** 10.7717/peerj.3732

**Published:** 2017-09-06

**Authors:** F. Joseph Pollock, Sefano M. Katz, Jeroen A.J.M. van de Water, Sarah W. Davies, Margaux Hein, Gergely Torda, Mikhail V. Matz, Victor H. Beltran, Patrick Buerger, Eneour Puill-Stephan, David Abrego, David G. Bourne, Bette L. Willis

**Affiliations:** 1 Australian Institute of Marine Science, Townsville, QLD, Australia; 2 AIMS@JCU, Townsville, Queensland, Australia; 3 ARC Centre of Excellence for Coral Reef Studies, James Cook University of North Queensland, Townsville, Queensland, Australia; 4 College of Science and Engineering, James Cook University of North Queensland, Townsville, Queensland, Australia; 5 Eberly College of Science, Department of Biology, Pennsylvania State University, University Park, PA, United States of America; 6 Centre Scientifique de Monaco, Monaco; 7 Department of Integrative Biology, University of Texas at Austin, Austin, TX, United States of America; 8 Department of Marine Sciences, University of North Carolina at Chapel Hill, Chapel Hil, NC, United States of America; 9 College of Natural and Health Sciences, Zayed University, Abu Dhabi, United Arab Emirates; 10 Department of Biology, Boston University, Boston, MA, United States of America

**Keywords:** Coral, Conservation, Spawning, Larvae, Restoration, Husbandry, Great Barrier Reef, Coral reef, *Symbiodinium*, *Acropora*

## Abstract

Here we describe an efficient and effective technique for rearing sexually-derived coral propagules from spawning through larval settlement and symbiont uptake with minimal impact on natural coral populations. We sought to maximize larval survival while minimizing expense and daily husbandry maintenance by experimentally determining optimized conditions and protocols for gamete fertilization, larval cultivation, induction of larval settlement by crustose coralline algae, and inoculation of newly settled juveniles with their dinoflagellate symbiont *Symbiodinium*. Larval rearing densities at or below 0.2 larvae mL^−1^ were found to maximize larval survival and settlement success in culture tanks while minimizing maintenance effort. Induction of larval settlement via the addition of a ground mixture of diverse crustose coralline algae (CCA) is recommended, given the challenging nature of *in situ* CCA identification and our finding that non settlement-inducing CCA assemblages do not inhibit larval settlement if suitable assemblages are present. Although order of magnitude differences in infectivity were found between common Great Barrier Reef *Symbiodinium* clades C and D, no significant differences in *Symbiodinium* uptake were observed between laboratory-cultured and wild-harvested symbionts in each case. The technique presented here for *Acropora millepora* can be adapted for research and restoration efforts in a wide range of broadcast spawning coral species.

## Introduction

Increased intensity and frequency of climate change events combined with increasing local anthropogenic pressures are responsible for alarming declines of coral reefs globally (Gardner et al., 2003; Schutte, Selig & Bruno, 2010; Burke, 2012; De’ath et al., 2012). Many reefs have now reached a point where they fail to recover naturally and risk shifting from coral to algal-dominated states (Hughes, 1994; Bellwood et al., 2004; Hughes et al., 2007; McClanahan et al., 2011). Novel research and management approaches are currently underway to better understand the drivers behind reef declines and to develop effective techniques to restore degraded reefs (Bellwood et al., 2004; Rinkevich, 2008; Rinkevich, 2014; Ateweberhan et al., 2013), but most reef restoration projects require the collection and fragmentation of sensitive, and in many cases protected, coral species from already ailing ecosystems. Alternative sources of coral recruits are required to overcome the logistical and ethical challenges such harvesting represents for coral researchers and reef managers.

Laboratory-reared coral juveniles provide environmentally responsible and easily-replicable alternatives to the fragmentation of wild-harvested adult colonies for restoration and research programs (Raymundo & Maypa, 2004; Petersen et al., 2005; Guest et al., 2014; Barton, Willis & Hutson, 2015). A single pair of broadcast-spawning adult corals can provide thousands of juveniles from one annual reproductive event (Harrison & Wallace, 1990; Baird, Guest & Willis, 2009). In addition, laboratory-cultured *Symbiodinium* strains or environmentally-sourced *Symbiodinoium* populations allow manipulation of algal symbiont clades in coral species through horizontal acquisition (i.e., symbionts are acquired from the environment rather than through vertical transmission from the parent). Laboratory-reared coral juveniles therefore present a promising resource opportunity for coral restoration, as well as for investigating processes influencing coral mortality during sensitive early life history stages (Vermeij, Fogarty & Miller, 2006; Randall & Szmant, 2009; Baria et al., 2010; Tebben et al., 2014; Dixon et al., 2015). Infection of coral juveniles with specific *Symbiodinium* strains has provided valuable insights into the role of symbiont clade in coral resilience and holds the potential to revolutionize coral restoration practices (Abrego et al., 2008; Littman, Bourne & Willis, 2010; Van Oppen et al., 2015). Recent escalation in the rates of coral decline in many parts of the world has resulted in increased focus on the potential for human-assisted coral evolution programs to produce more tolerant coral-*Symbiodinium* genotypes (Van Oppen et al., 2015). From a restoration perspective, the introduction of captive-reared offspring of broadcast-spawning corals onto reefs provides a mechanism to increase genetic diversity, improve early life history survival and introduce resilient genotypes onto damaged reefs (Van Oppen & Gates, 2006; Nakamura et al., 2011; Baria et al., 2012; Guest et al., 2014; Rinkevich, 2014; Van Oppen et al., 2015).

While sexually-derived coral juveniles have clear benefits for reef restoration and research, the infrequent nature of coral spawning events and the sensitivity of coral larvae have historically rendered the culturing of coral juveniles a time- and labor-intensive exercise. While previous studies have focused on improving techniques for effective delivery of reared juveniles onto reefs (Guest et al., 2014), relatively few have developed optimized techniques for the fragile aspect of gamete release, fertilization, larval rearing and settlement (Vermeij, Fogarty & Miller, 2006). Here we optimize rearing techniques for *Acropora millepora,* an emerging model coral species (Miller & Ball, 2000) and ecologically-important reef builder, to determine (1) optimal larval rearing densities to maximize yield per unit effort, (2) optimal settlement cue species and dosage, and (3) optimal *Symbiodinium* strain and inoculation density. We describe a simple and effective method to mass culture coral larvae that can be directly applied for coral research and restoration.

## Materials and Methods

### Coral spawning, gamete fertilization and larval rearing

**Figure 1:**
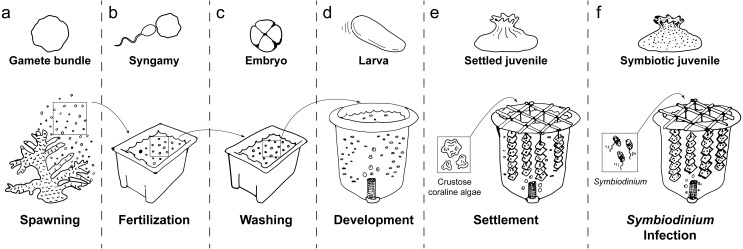
Schematic of coral rearing process. (A) Prior to spawning, individual coral colonies are isolated in 70 L plastic bins. Following gamete release, egg/sperm bundles are immediately collected from the water surface and (B) mixed with those from other colonies in clean plastic bins to allow fertilization. (C) One hour after observation of the first embryo cleavage, embryos are processed through three consecutive washing steps to remove excess sperm and decrease polyspermy. (D) At the 2- to 4-cell stage of development, embryos are transferred to aerated 420 L flow-through larval culture tanks. Once fully developed, larvae begin exhibiting settlement competency behavior (i.e. substratum searching), (E) ground crustose coralline algae is added to induce larval settlement. Following settlement onto the settlement substrate (e.g., terracotta tile), (F) *Symbiodinium* are added to rearing tanks to initiate symbiosis. Symbiotic, settled juveniles are then ready for downstream use in research and/or restoration programs.

Mature, gravid colonies of the hermaphroditic broadcast spawning coral *Acropora millepora* were collected from reef crests (4 to 6 m) at Orpheus and Pelorus Islands in the central Great Barrier Reef (GBR), five days prior to predicted spawning dates in November 2011 and 2012 (Great Barrier Reef Marine Park Authority permit number G10/33312.1). Reproductive maturity was verified prior to harvesting, as confirmed by the presence of pigmented oocytes in test branches subsampled from mature regions of colonies (Wallace, 1985). Harvested colonies were transported by boat (in covered 70 L plastic bins) to Orpheus Island Research Station (OIRS), where they were transferred to 1,000 L plastic flow-through tanks supplied with 1 µm filtered seawater (FSW) at ambient sea temperature (28°C). Seawater was filtered through commercially-available 10″ poly-spun polypropylene sediment filters held in Aqua-Pro HD1020 housings. Corals were provided with continuous aeration via air stones. At dusk on predicted nights of spawning (approximately 2 h before predicted spawning time), colonies were placed in individual 70 L plastic bins without water flow or aeration. Following gamete release, gamete bundles were immediately skimmed from the water surface with minimal seawater (Fig. 1A) and gently mixed with those from seven other colonies in clean (i.e., pre-bleached) 70 L fertilization tanks filled with 0.5 µm FSW (Fig. 1B). Fertilization was verified when the initial cleavage furrow was observed by microscopy (approximately 1.5 h after fertilization) and embryos were allowed to develop for one additional hour. Embryos were then gently washed via three consecutive transfers (via pre-bleached plastic beakers) into clean 70 L tanks in order to remove excess sperm and minimize polyspermy of yet unfertilized eggs (Fig. 1C). Embryo transfers during washing involved minimal agitation to avoid fragmenting early stage embryos.

Washed embryos were transferred into 420 L larval culture tanks (Fig. 1D) filled with ultra-violet irradiated, 0.5 µm FSW by 4 h after spawning. Water flow and aeration were turned off while embryos developed through fragile multicellular stages to the early gastrula stage. Water flow (1 L per minute, enabling >3 full water exchanges per day) and aeration were turned on approximately 3.5 and 20 h, respectively, after transfer, and larvae were maintained at 28°C under 12 h:12 h light:dark cycles (∼100 pmol photons m^−2^ s^−1^). Larval culture tanks were round to minimize stagnant areas and fitted with a central drain. In each tank, the central drain was covered with a plankton mesh filter to prevent loss of embyros and larvae, and connected to an external standpipe to control water level within the tank (Fig. S1A). A circular air stone at the base of each filter provided a curtain of bubbles that prevented embryos and larvae from exiting with the outflow. Embryonic development was monitored microscopically until ciliated, motile planulae had formed (∼48 h post-fertilization).

In the following assays, swimming planulae were used to experimentally determine optimal larval rearing conditions, including larval stocking densities (see ***Stocking density optimization***, Fig. 1D), settlement cues (see ***Settlement cue optimization***, Fig. 1E), and *Symbiodinium* infections conditions (see ***Symbiodinium*** ***infection optimization***, Fig. 1F), as described below.

### Stocking density optimization

To determine the optimal larval stocking density to maximize rates of larval survival and settlement, coral larvae were haphazardly partitioned across six 420 L culture tanks, yielding two experimental replicates at each of three stocking densities (1 [“high”], 0.5 [“moderate”] and 0.2 larvae mL^−1^ [“low”]). Each culture tank was equipped with a flow-through seawater system and aeration, as described above. Larval density was quantified every 24 h for six days. At each time point, culture tanks were stirred with pre-bleached spatulas to evenly distribute coral larvae throughout the water column, and five replicate 150 mL water samples were collected from midwater within each tank. The number of live larvae in each sample was quantified using a Bogorov counting chamber and a dissecting microscope.

Larval settlement success was also compared among the three stocking density treatments. A subsample of larvae from each tank was checked daily for settlement competency. When larvae began searching the substratum for settlement sites (seven days post-spawning), 48 pre-conditioned (three months in the field) and autoclaved (to prevent the introduction of potential pathogens) terracotta settlement tiles (11 ×11 ×1 cm) were added to each tank. Eight settlement tiles were suspended on each of six strings per tank, with 3 cm long plastic spacers separating consecutive tiles (Fig. 1E, Figs. S1B, S1C). Approximately 50 mg of autoclaved crustose coralline algae (CCA) slurry (see ***Settlement cue optimization***) was placed on each tile to induce settlement. Five days after the introduction of settlement tiles and CCA, a census of up to 5-day old recruits was conducted by examining all surfaces of all settlement tiles with a dissecting microscope.

To quantify the number of settled larvae per unit effort at each stocking density, detailed records of maintenance effort were kept for each tank throughout the rearing process. Tanks were monitored every 3 to 6 h and maintenance was conducted as needed to ensure high water quality. Organic matter and lipid aggregations from non-viable eggs and embryos were removed using clean beakers and paper towels skimmed lightly over the water surface or around the sides of tanks at the water line when required. Clean pipettes were also used to remove smaller aggregates. Patches of organic matter accumulating on the bottom and sides of tanks were removed via suction through bleach-sterilized and FSW-rinsed 12 mm diameter rubber hoses. Unit effort was calculated by summing the time (in person-hours) taken to maintain each culture tank, during the larval maturation period. Yield per unit effort was calculated by dividing the number of coral recruits (determined by dissecting microscope census) in each tank by the unit effort for that tank.

### Crustose coralline algae strain optimization

Crustose coralline algae (CCA) are known to induce metamorphosis and settlement of coral larvae (Heyward & Negri, 1999; Harrington et al., 2004; Tebben et al., 2015), but methods for preparing CCA samples and inoculating larval cultures are less well-studied. To identify an effective method for inducing larval settlement in the laboratory, settlement rates of *A. millepora* larvae were quantified across a range of phenotypically diverse CCAs and preparation methods. CCA fragments with attached microbial communities (as determined through genotyping; see *Taxonomic characterization of CCA communities* below) were collected from Pelorus Island using a hammer and chisel prior to coral spawning in November 2011 (CCA fragments 1–8) and 2012 (CCA fragments 9–14) (Fig. S2). CCA fragments were maintained in a 1,000 L flow-through tank at OIRS and finely ground using a sterilized mortar and pestle prior to inoculation.

In 2011, larval settlement was tested in response to eight CCA fragments (labeled 1–8) and three CCA preparation treatments. Each of the CCA preparation treatments combined subsamples of all eight CCA fragments in roughly equal proportions, and were prepared as (a) “Unwashed” (labeled U) CCA fragments neither washed nor autoclaved prior to inoculation, (b) “Washed” (labeled W) CCA fragments washed five times with FSW, but not autoclaved prior to inoculation, and (c) “Autoclaved” (labeled A): CCA fragments washed five times with FSW and autoclaved prior to inoculation.

In 2012, induction of larval settlement was tested in response to six CCA fragments (labeled 9–14) and a CCA preparation treatment involving ethanol extraction of subsamples from all six fragments to control for cue dosage. CCA fragments were washed five times with FSW but not autoclaved, as no differences in settlement behavior were detected between “U”, “W” or “A” preparation treatments in 2011. For the ethanol-extraction preparation, subsamples of all six fragments were combined in equal proportions and left in an equal volume of 100% ethanol in a 50 mL conical tube for 24 h. The resulting ethanol supernatant was collected and stored at 4°C until used in inoculations.

All settlement trials were conducted in sterile 6-well plates, with each well receiving 10 mL of FSW, a single drop (∼10 mg CCA) of CCA slurry (i.e., CCA ground with mortar and pestle in FSW), or 20 µl EtOH extract, and 20 competent *A. millepora* larvae. For the ethanol-extracted sample, each settlement assay well received 20 µl of the extract, the ethanol was allowed to evaporate, and then 10 mL of FSW was added to each well. Each CCA treatment was replicated six times and treatments were randomly assigned across plates. FSW control treatments were also included (6 replicates per year); settlement was never observed in control treatments. The proportion of metamorphosed larvae (i.e., those showing visible septa) was quantified after 24 h in 2011 and 60 h in 2012 using a fluorescent stereomicroscope MZ-FL-III (Leica, Bannockburn, IL, USA) equipped with F/R double-bandpass filter (Chroma no. 51004v2).

### Taxonomic characterization of CCA communities

Taxonomic compositions of assemblages associated with each CCA fragment were determined via deep metabarcoding amplicon sequencing with modifications for 454-rapid technology (as per Davies et al., 2013; Davies et al., 2014; see Supplemental Information 1). Sequences were trimmed and analyzed as described previously (Davies et al., 2014). In brief, raw reads were split by barcode (Table S1), adaptors were trimmed, and bases of low quality and reads <250 bp were discarded. *cd-hit-454* (Huang et al., 2010) clustered filtered reads at 0.97 and then clusters containing >1% of filtered reads chosen as distinct operational taxonomic units (OTUs). Independent sample sequences were then mapped to reference OTUs using the *runMapping* module of Newbler v. 2.6 (Roche) with repeat score threshold (parameter –rst) of 3. Proportions of reads uniquely mapping to an OTU were considered to be the OTU relative abundances in each CCA assemblage. OTUs accounting for the greatest number of mapped reads in an assemblage were assigned to taxonomic Order based on BLAST matches (Altschul et al., 1997) against nonredundant (nr) NCBI database.

### CCA dosage experiments

To determine the optimal quantity of CCA required to induce larval settlement for the coral *A. millepora*, three concentrations of autoclaved CCA slurry were tested in 2011. A slurry of all eight CCA fragments was used, as described above for the autoclaved preparation. For larval settlement trials, each well of three sterile 6-well plates received 10 mL of FSW and 10 competent larvae (i.e., larvae exhibiting substratum-searching behavior, detected four days post-spawning). Wells were then randomly assigned to one of the following three CCA treatments: 0 mg, 10 mg, or 50 mg of autoclaved and FSW washed CCA slurry per well (*n* = 60 larvae per treatment). Larval behavior and condition were assessed 48 h after CCA addition and classified as belonging to one of the following four categories: unmetamorphosed (i.e., planula stage), metamorphosed and not attached, metamorphosed and attached (i.e., settled), or dead.

### *Symbiodinium* isolation culturing

Axenic cultures of C1 and D *Symbiodinium* were maintained in growth medium comprised of a modified F/2 and Erdschreiber medium (Guillard & Ryther, 1962). Briefly, seawater was supplemented with 4 mg L^−1^ Na_2_HPO_4_, 1 g l^−1^ NaNO_3_, 1 mL l^−1^ from a 1,000X concentrated A_5_+CO micronutrient solution (Sussman et al., 2009), 2.5 mg l^−1^ GeO_2_, 80 mg l^−1^ G-penicillin, 80 mg l^−1^ streptomycin, 40 mg l^−1^ amphotericin, 0.4 mg l^−1^ thiamine-HCl, 2 µg l^−1^ biotin, and 2 µg l^−1^ vitamin B12 (cyanocobalamin). The growth medium was 0.22 µm filtered and stored at 4°C in the dark. Before growth medium was used, the 0.22 µm filtration step was repeated. *Symbiodinium* cultures were maintained at 28°C under 12 h:12 h light:dark cycle (120 pmol photons m^−2^ s^−1^). Freshly-isolated C1 *Symbiodinium* were obtained from *Acropora tenuis* collected from Nelly Bay, Magnetic Island (central Great Barrier Reef). Tissue was airbrushed, collected in 5 µm FSW and homogenized for 1 m (IKA T10 Basic homogenizer, Malaysia). Homogenate was filtered twice through four layers of 10 µm plankton mesh. *Symbiodinium* cells were spun down for 5 min at 3,000× g and washed three times with 5 µm FSW. The number of *Symbiodinium* cells per ml was quantified (*n* = 10) using a Neubauer hemocytometer. Symbiont genotype was confirmed by single-stranded conformation polymorphism (SSCP) analysis of ITS1 PCR amplicons using reference samples of known genotypes, as described by Van Oppen et al. (2001).

### *Symbiodinium* infection optimization

To determine the optimal clade, source and density of *Symbiodinium* cells for symbiont uptake, settled juvenile of the coral *A. millepora* were exposed to either laboratory-cultured or freshly-isolated suspensions of *Symbiodinium* C1, or to laboratory-cultured clade D *Symbiodinium*. Swimming coral larvae were settled in sterile six-well plates at a density of 10 larvae per well (each well containing 10 mL 0.2 µm FSW) by adding 10 mg of autoclaved, pooled CCA slurry to each well, as described above. Wells were then randomly assigned to one of the six *Symbiodinium* treatments: laboratory-cultured C1 *Symbiodinium* at 10^2^, 10^4^ or 10^6^ cells mL^−1^ FSW; laboratory-cultured clade D *Symbiodinium* at 10^4^ cells mL^−1^ FSW; freshly-isolated C1 *Symbiodinium* at 10^4^ cells mL^−1^ FSW; or FSW negative control. This experimental design allowed for simultaneous assessment of the influence of *Symbiodinium* density (within clade C1) and direct comparison of clades C1 and D at the same stocking density (10^4^ cells mL^−1^). Six replicate wells (each containing 10 settled larvae) were inoculated for each treatment (*n* = 6 replicates; 10 settled polyps per replicate). *Symbiodinium* uptake was quantified 48 h after symbiont addition by sacrificing approximately three randomly-selected juveniles from each well (*n* = 16 to 24 juveniles per treatment). Juveniles were rinsed in FSW and algal symbionts harbored within their tissues were counted using light and fluorescence microscopy.

### Statistical analysis

One-way analysis of variance (ANOVA) was employed to assess the effect of larval stocking density on both larval settlement rate and settlement per unit effort on day 5 after the addition of settlement tiles. One-way ANOVA was also employed to assess the effect of CCA dosage and community on larval settlement behavior. Data were tested for normality and homoscedasticity (Shapiro–Wilk and Bartlett tests, respectively) prior to performing statistical analyses. Data not satisfying these criteria were power transformed using the Box–Cox method to meet parametric criteria of normality and equal variances. Post hoc comparisons between groups for all ANOVA tests were performed using Tukey’s honestly significant difference (HSD) test. The non-parametric Kruskal–Wallis test was employed to assess the effect of *Symbiodinium* treatments on algal uptake at 48 h (these data did not meet ANOVA assumptions, even after Box–Cox transformation), and post hoc comparisons between groups were performed using Dunn’s multiple comparisons test. To assess the effect of larval stocking density and time on surviving larval density, a Generalized Additive Model (GAM), more specifically a Poisson Regression analysis, was employed with density, time, and tank included as fixed factors (density * time + tank). This model also incorporated an assessment of temporal auto-correlation with an Auto-Regressive Moving Average (ARMA) model implemented in the R package ‘mgcv’ (Wood, 2016). All statistical analyses were performed using R: Statistical Computing Software (R Development Core Team, 2015).

## Results

### Coral larval density optimization

**Figure 2:**
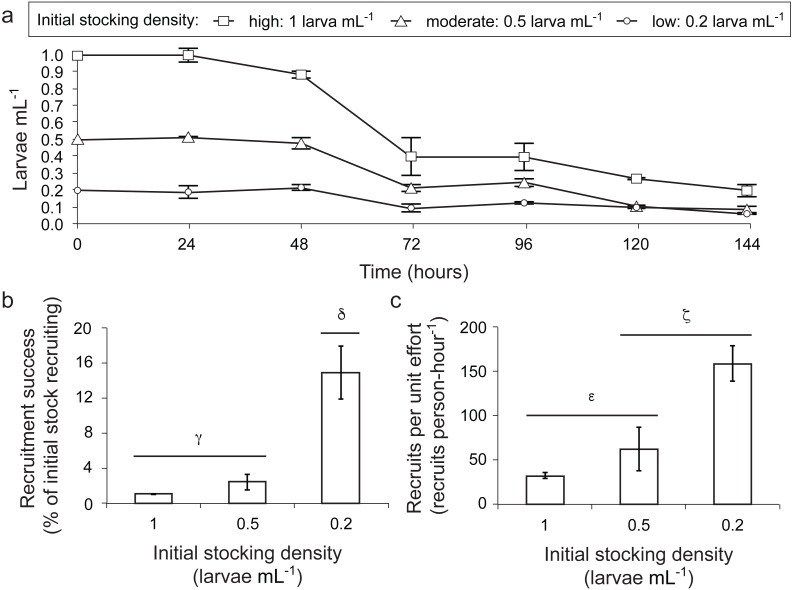
Influence of larval stocking density (1 [high], 0.5 [moderate] and 0.2 larvae ml^−1^ [low]) on density of surviving larvae, recruitment success and recruitment yield per unit effort for the coral *A. millepora*. (A) Density of surviving larvae in culture tanks (larvae ml^−1^) over the 144 h trial; (B) recruitment success (% of initial stock settling on terracotta tiles and alive 5 days after tiles were deployed in culture tanks); and (C) recruitment per unit effort (number of larvae settling on terracotta tiles and alive at day 5 per person hour effort). The five day settlement/recruitment experiment followed the 144 h survival trial. Error bars show standard error; Greek symbols indicate homogenous post hoc groupings (Tukey’s HSD *P* < 0.05) (*n* = 2 × 420 L larval culture tanks per treatment).

Regression analysis (model fit: adjusted *R*^2^ = 0.955) indicated that time (${\chi }_{5,35}^{2}=\text{7}80,130$; *P* < 0.001) and stocking density (${\mathrm{\chi }}_{2,35}^{2}=\text{282,181}$; *P* < 0.001) significantly influenced larval mortality (Fig. 2A). This analysis also revealed a significant interaction between time and larval density (${\mathrm{\chi }}_{10,35}^{2}=\text{72,962}$; *P* < 0.001). In addition, we found a significant effect of experimental tank (${\mathrm{\chi }}_{3,35}^{2}=\text{11,521}$; *P* < 0.001), which can be attributed to our relatively low number of replicate culture tanks (*n* = 2 per stocking density). Overall, clear trends in the mortality rates of larvae were observed: mortality rates of those held at the lowest stocking density (0.2 larvae mL^−1^) were significantly lower than those held at moderate (0.5 larvae mL^−1^) and high (1 larvae mL^−1^) densities (Fig. 2A). Mortality rates in moderate stocking density tanks were also lower than those in high stocking density tanks (Fig. 2A). In all treatments, mortality was particularly high during the 48–72 h and the 96–120 h periods (Fig. 2A). While larval densities in the high stocking density tanks remained higher compared to low and moderate stocking density tanks throughout the experiment, larval densities in the moderate stocking density tanks dropped below larval densities in low stocking density tanks after 120 h (Fig. 2A). Ultimately, this resulted in an average survival of 44.5% (± 9.3) of the larvae in the low stocking density tanks versus only 12.4% (±0.8) and 20.1% (±3.7) in moderate and high density tanks, respectively, after 144 h.

Both density of larvae surviving until settlement (*F*_2,3_ = 28.02, *P* < 0.012, Fig. 2B) and yield per unit effort (*F*_2,3_ = 10.78, *P* = 0.043, Fig. 2C) differed significantly among larval stocking densities. Larvae reared at the lowest density had 15 times greater settlement success (average ± SE: 14.9% ± 3.0% of initial stock settling; Tukey’s HSD, *P* = 0.011) and yielded nearly five times more recruits per unit effort (158.4 ± 20.1 recruits per person-hour; Tukey’s HSD, *P* = 0.040) than those at the highest stocking density (1.0% ± 0.0% of initial stock settling; 32.1 ± 3.0 recruits per person-hour) (Figs. 2B and 2C). Recruitment success in the lowest stocking density treatment was also 6 times higher than in the moderate density treatment (2.4% ± 0.9% recruitment; Tukey’s HSD, *P* = 0.030), although yield per unit effort did not differ significantly between low and moderate density treatments (Tukey’s HSD, *P* = 0.108; Figs. 2B and 2C).

**Figure 3:**
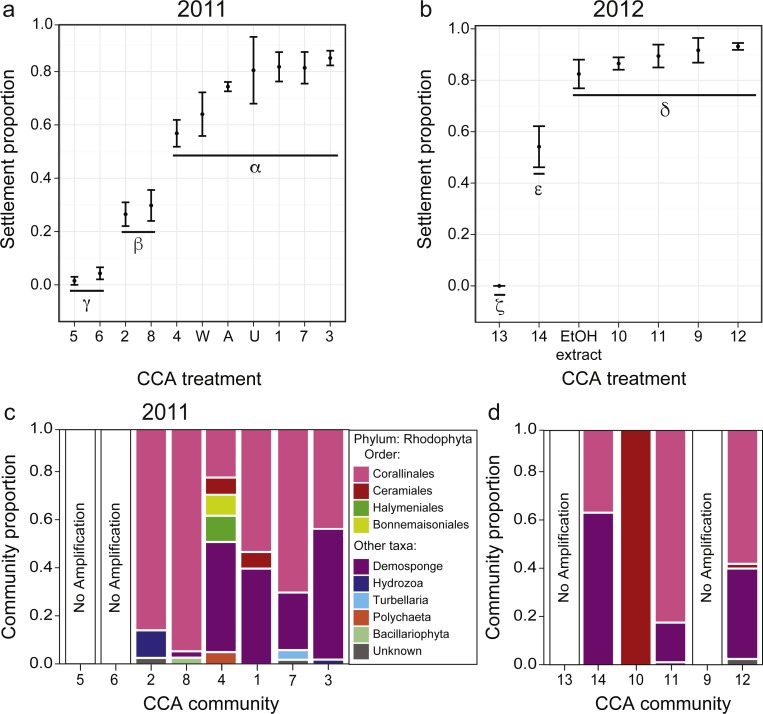
Larval settlement in response to assemblages associated with CCA fragments for the coral *A. millepora*. Settlement response trials in: (A) 2011, which tested assemblages associated with eight phenotypically distinct CCA fragments (1–8), and three CCA preparation treatments: pooled assemblages from all 8 fragments that were either unwashed (“U”, unwashed), washed five times with fresh seawater (“W”, washed), or washed and autoclaved (“A”, autoclaved); and (B) 2012, which tested assemblages associated with six phenotypically distinct CCA fragments (9–14), and a CCA preparation treatment involving ethanol precipitation of pooled samples to control for cue dosage. Error bars show standard error; Greek symbols indicate homogenous post hoc groupings after multiple test correction (Tukey’s HSD *P* < 0.05) (*n* = 6 replicates per treatment; 20 larvae per replicate). Relative proportions of mapped reads belonging to various taxonomic Orders within the Phylum Rhodophyta (pink, red, green, yellow) and non-Rhodophyta taxa associated with CCA fragments tested in (C) 2011, and (D) 2012.

### Settlement cue optimization

#### CCA community

*Acropora millepora* larvae exhibited distinct preferences for specific CCA cue assemblages in both 2011 and 2012 (*P* < 0.001). No larvae were ever observed to settle in control wells. In 2011, of the eight phenotypically diverse CCA fragments tested, *A. millepora* larval settlement was highest in response to assemblages associated with CCA fragments 1, 3, and 7 (Fig. 3A, *P* < 0.001, Fig. S2). Larvae did not discriminate among the three CCA preparation treatments, and settlement in the pooled mixtures was statistically similar to the most preferred cues (i.e., assemblages associated with CCA fragments 1, 3, 7; Fig. 3A). CCA fragments 5 and 6 were least effective in inducing settlement in 2011. When samples were prepared for sequencing, CCA fragments 5 and 6 failed to amplify, suggesting that they were associated with highly divergent assemblages or that PCR was inhibited. Assemblages of all 2011 CCA fragments successfully amplified contained the Order Corallinales (the order to which CCAs belong) BLAST hits (Fig. 3C); however, the presence of Corallinales reads within assemblages did not guarantee successful *A. millepora* settlement (Fig. 3C).

In 2012, of the six phenotypically diverse CCA fragments (CCA numbers 9–14, Fig. S2) tested, larvae exposed to assemblages associated with CCA fragments 9, 10, 11 and 12, as well as the ethanol extract of pooled assemblages, exhibited the highest settlement rates. Assemblage 14 also induced high, but significantly lower, rates of settlement (Fig. 3B, Fig. S2). The success of the ethanol extract is of particular interest, given that use of extracts allow for greater control for both cue dosage and composition. CCA fragment 13 failed to induce settlement and again, it was among the two samples (9, 13) that failed to amplify during sample preparation for sequencing. Sequencing results from 2012 also demonstrated that most CCA fragment assemblages had reads with high sequence similarity to the Order Corallinales. However, the assemblage associated with CCA fragment 10, which only returned sequences corresponding to the Order Ceramiales, was also an effective settlement inducer (Fig. 2D).

**Figure 4:**
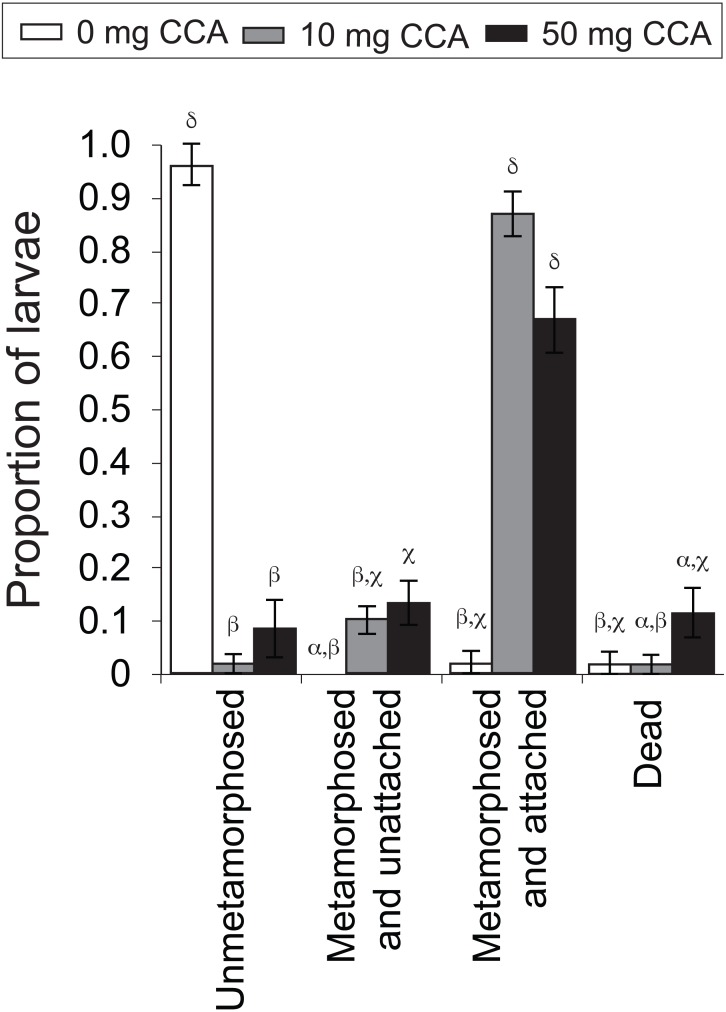
Average proportion of larvae of the coral *A. millepora* displaying one of four settlement stages or health states (un-metamorphosed, metamorphosed and unattached, metamorphosed and attached, or dead) 48 h after the addition of washed and autoclaved CCA from 2011 pooled samples, at three concentrations: 0 mg (white), 10 mg (gray), 50 mg (black) per 10 ml filtered seawater. Error bars show standard error; Greek symbols indicate homogenous post hoc groupings (Tukey’s HSD *P* < 0.05) (*n* = 6 replicates per treatment; 10 larvae per replicate).

#### CCA dosage

CCA dosage significantly influenced rates of larval metamorphosis and settlement (*F*_11,56_ = 81.28, *P* = 0.000, Fig. 4). At the end of the 48 h settlement trial, there was a significantly higher rate of settlement (scored as “metamorphosed and attached”; Fig. 4) for larvae exposed to the moderate (10 mg in 10 mL FSW; average ± SE: 86% ± 4%) and high (50 mg; 70% ± 8%) CCA dosages, in comparison to those exposed to the control treatment (0 mg; 2% ± 2%) (*P* = 0.000, Fig. 4). Nearly all larvae that were not exposed to a CCA cue (i.e., control treatment) remained in the planula stage (i.e., unmetamorphosed) (96% ± 4%, Fig. 4). Larval mortality rates were highest in the high CCA dosage treatment (12% ± 5%), but statistically, they were not significantly greater than the moderate dosage (2% ± 2%) and control treatments (2% ± 2%) (*P* > 0.05, Fig. 4).

### *Symbiodinium* infection optimization

**Figure 5:**
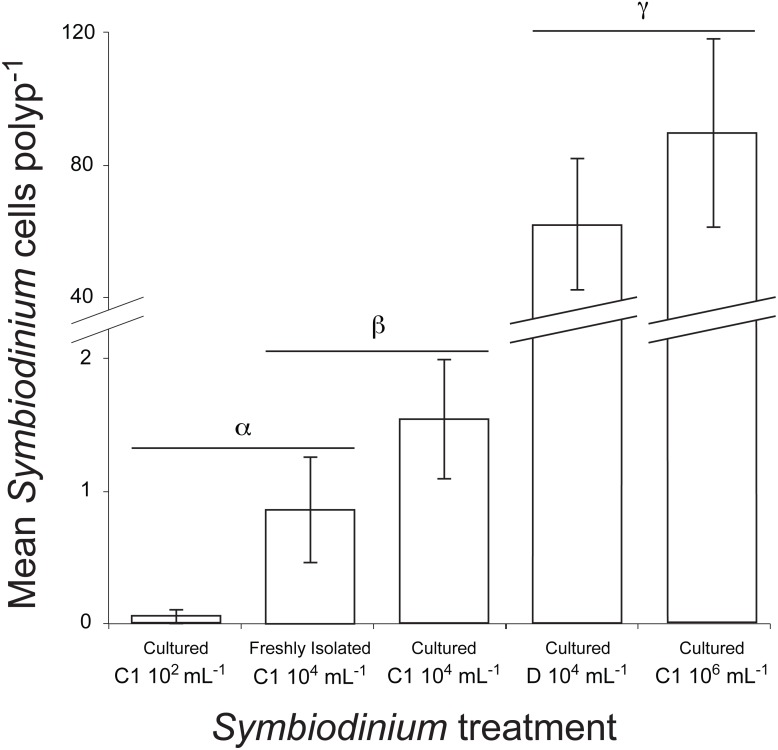
Mean number of *Symbiodinium* cells per polyp at 48 h post inoculation in juvenile recruits of the coral *A. millepora* inoculated with one of five treatments: laboratory-cultured C1 *Symbiodinium* at 10^2^, 10^4^ or 10^6^ algal cells ml^−1^; or freshly-isolated C1 *Symbiodinium* at 10^4^ cells ml^−1^; or laboratory-cultured clade D *Symbiodinium* at 10^4^ cells ml^−1^. No *Symbiodinium* were ever observed in un-inoculated juvenile recruits (i.e., negative control), therefore this treatment is not shown. Error bars show standard error; Greek symbols indicate significant post hoc groupings (Dunn’s multiple comparisons test, *P* < 0.05) (*n* = 16 to 24 juveniles per treatment).

Symbiont uptake rates differed significantly among *Symbiodinium* inoculation densities and clades (*df* = 4, Kruskal–Wallis chi-squared =92.216, *P* = 0.000, Kruskal–Wallis, Fig. 5). At the end of the 48 h trial, symbiont levels were highest in juveniles inoculated with laboratory cultures of either C1 *Symbiodinium* at 10^6^cells mL^−1^ (90 ± 28 *Symbiodinium* cells per polyp) or clade D *Symbiodinium* at 10^4^ cells mL^−1^ (62 ± 20 *Symbiodinium* cells per polyp) (*P* < 0.01). Despite the 100-times lower inoculation dose of clade D symbionts, there was no significant difference in *Symbiodinium* uptake between these treatments using lab-cultured symbionts (*P* > 0.05, Fig. 5). *Symbiodinium* uptake in all other treatments was relatively low (i.e., <2 cells per polyp on average). Symbiont uptake did not differ significantly between cultured (1.5 ± 0.5) and freshly-isolated (0.9 ± 0.4) C1 symbionts inoculated at 10^4^ cells mL^−1^, or between freshly-isolated C1 symbionts inoculated at 10^4^ cells mL^−1^ and cultured C1 symbionts inoculated at 10^2^ cells mL^−1^ (0.1 ± 0.1) (*P* > 0.05, Fig. 5). However, uptake of cultured C1 *Symbiodinium* was significantly higher when juveniles were exposed to 10^4^ *Symbiodinium* cells mL^−1^ than at 10^2^ cells mL^−1^ (*P* < 0.05, Fig. 5). No *Symbiodinium* cells were ever observed in juveniles not inoculated (i.e., negative control).

## Discussion

With coral reefs worldwide facing mounting pressures from local and global stressors (De’ath, Lough & Fabricius, 2009; Schutte, Selig & Bruno, 2010), the demand for coral larvae and juveniles to help experimentally identify factors contributing to reef declines, and for the purpose of restocking degraded reefs is increasing. Here we describe a simple, yet effective method for rearing sexually-derived coral larvae for downstream use in coral reef restoration and scientific research. Relatively few publications offer specific guidelines for rearing and maintaining corals produced by sexual reproduction despite the many advantages of this approach over asexual propagation. Sexual propagation: (1) results in higher genetic diversity than asexual propagation; (2) exploits corals’ high fecundity (i.e., one sexually mature broadcast spawning colony can produce hundreds of thousands of eggs from a single spawning event); (3) has minimal impact on existing reefs, because parent colonies can be returned to the reef after spawning; and (4) allows for bespoke host-symbiont combinations in coral species with horizontal *Symbiodinium* transmission (Edwards, 2010; Van Oppen et al., 2015). The methods described here were optimized for *A. millepora*, but this approach could be readily adapted to other broadcast spawning coral species.

### Stocking density optimization

Choosing an appropriate larval stocking density is essential to maximize survival during the vulnerable period leading up to settlement and metamorphosis. Here we demonstrate that low stocking densities (i.e., 0.2 larvae mL^−1^) result in lower mortality and maximize survival and settlement success, while minimizing maintenance effort. These results indicate that lower stocking densities, preferably at or below 0.2 larvae mL^−1^, maximize larval survivorship and recruitment and minimize maintenance effort. These findings are consistent with the best practice guidelines presented in Edwards (2010), which advise not exceeding 0.3 larvae mL^−1^. Even at a stocking density of 0.2 larvae mL^−1^, a single 420 L tank can yield well over 11,000 coral recruits for downstream use.

### Settlement cue optimization

Certain species of CCA are known to induce settlement and metamorphosis in coral larvae, but not all species and/or strains elicit equal settlement success (Morse et al., 1996; Heyward & Negri, 1999; Harrington et al., 2004; Davies et al., 2014). Here we demonstrate that while several CCA assemblages failed to stimulate larval settlement, the presence of these non-settlement-inducing assemblages did not inhibit settlement if other suitable assemblages were present. This observation suggests that mixtures comprised of a diverse array of CCA assemblages are a practical option for inducing larval settlement in the field. This approach is particularly appealing given the challenging nature of *in situ* CCA identification. The genomics technique employed here aimed to identify these CCA assemblages *post hoc*, however this technique failed to resolve the desired species-level CCA designations, highlighting the need for a more robust and curated sequence database of CCAs for future identification studies.

Selecting an appropriate CCA delivery method is also important for promoting settlement. We found that ethanol extracts of CCA mixtures and direct CCA addition were equally effective for settlement induction. Given that CCA dosage can impact larval settlement and mortality rates, the ability to add a precise quantity of CCA via bulk ethanol extraction provides an appealing option to control the timing, location, and, to some extent, quantity of settling larvae. However, each novel ethanol extract will be unique in its assemblage and cue concentration. This method is therefore only quantitative with respect to larval settlement within a single ethanol extract. While coral juveniles in this study were settled onto terracotta tiles, a wide variety of alternative settlement substrates are available to suit specific research and restoration applications (reviewed in Edwards, 2010).

### *Symbiodinium* infection optimization

The controlled introduction of specific *Symbiodinium* species/strains into juvenile corals that acquire their symbionts via horizontal transmission provides an important nutritional boost to young corals and facilitates the creation of customized host-symbiont combinations. Custom coral-algal combinations provide a powerful tool for investigations into the drivers of symbiosis breakdown (i.e., bleaching) and for the introduction of more stress tolerant holobionts onto reefs (Abrego et al., 2008; Van Oppen et al., 2015). Here we demonstrate significant differences in infectivity among *Symbiodinium* clades, with Clade D *Symbiodinium* reaching an order-of-magnitude greater density than Clade C symbionts inoculated at the same concentration. It should be noted that rearing conditions (e.g., light and temperature) can significantly influence *Symbiodinium* uptake dynamics and that *Symbiodinium* infectivity varies greatly among coral host/symbiont combinations (Mieog et al., 2009; Abrego, Willis & Van Oppen, 2012). Both *Symbiodinium* strains employed in this study are known to infect *A. millepora* (Mieog et al., 2009; Abrego, Willis & Van Oppen, 2012), but detailed information on *Symbiodinium* infectivity and the long-term fidelity of resulting symbioses in less well-studied coral species is sorely lacking (Weis et al., 2001; Baird et al., 2007). In this study, *Symbiodinium* were offered at only a single time point, which may have limited *Symbiodinium* uptake. Multiple, consecutive rounds of *Symbiodinium* addition could enhance symbiont uptake in compatible host/symbiont pairs, and this approach should therefore be considered when feasible (Abrego, Willis & Van Oppen, 2012). Further experimentation into the uptake dynamics and fidelity of specific coral-*Symbiodinium* combinations is required to better understand their feasibility, costs and benefits.

This study found no significant differences in *Symbiodinium* uptake between laboratory-cultured and wild-harvested Clade C symbionts. Symbiont cultures grown in the laboratory can yield large volumes and densities of *Symbiodinium* from small starter cultures that can be reused and even shared among laboratories with a minimal sacrifice of corals collected from reefs. However, the use of diverse *Symbiodinium* strains collected from wild populations allows for the cultivation of naturally-occurring adaptive variation. Researchers and restorations managers must therefore select *Symbiodinium* collection/cultivation strategies most closely aligned with their objectives.

## Conclusions

The combined pressures of local pollution, overexploitation and global climate change have led to dramatic declines in coral cover and pronounced shifts in community composition on many coral reefs worldwide (Bruno & Selig, 2007; De’ath et al., 2012). Faced with the possibility that corals will be unable to naturally withstand these pressures, researchers and reef managers have begun exploring the utility of active coral restoration (Hein et al., 2017; Rinkevich, 2014). Most coral restoration projects currently rely upon the propagation and outplanting of asexually derived coral clones produced via fragmentation of adult coral colonies (Young, Schopmeyer & Lirman, 2012; Ng & Chou, 2014). These techniques are capable of producing large coral biomass, but the limited genetic diversity of outplanted clones could hinder their capacity to adapt to rapidly changing environmental conditions (Van Oppen et al., 2015). The use of sexually-derived corals and even custom coral-*Symbiodinium* combinations has been suggested as a means to bolster and even augment corals’ capacity to adapt to rapidly changing environments (Van Oppen et al., 2015).

In this study, we describe a simple and effective technique for rearing sexually-derived coral propagules from spawning through to settlement, and we provide optimized conditions for larval stocking density, CCA addition and *Symbiodinium* inoculation in a broadcast spawning species of *Acropora*. This protocol maximizes larval survival and minimizes expense, effort and impact on natural coral populations. While this study focused on *A. millepora*, the techniques described here can inform research and restoration efforts in a wide range of broadcast spawning coral species.

##  Supplemental Information

10.7717/peerj.3732/supp-1 Supplemental Information 1 Supplemental Materials Supplementary Materials and Methods, Supplementary Figures, and Supplementary Table

## Additional Information and Declarations

### Field Study Permissions

The following information was supplied relating to field study approvals (i.e., approving body and any reference numbers): This work was approved by the Great Barrier Reef Marine Park Authority (permit number G10/33312.1).

### DNA Deposition

The following information was supplied regarding the deposition of DNA sequences: All sequence files have been deposited in the National Center for Biotechnology Information’s BioProject archive under accession number PRJNA331102.

### Data Availability

The following information was supplied regarding data availability: Pollock, Frederic (2017): Coral larvae rearing for restoration and research. figshare.
https://dx.doi.org/10.6084/m9.figshare.4737844.v1.
