# Supplementary material for: Coral larvae for restoration and research: a large-scale method for rearing Acropora millepora larvae, inducing settlement, and establishing symbiosis
Source: PeerJ. 2017 Sep 6;5:e3732. doi: 10.7717/peerj.3732 (PMC5591638; doi:10.7717/peerj.3732)
Supplement: Supplemental Information 1 — Supplementary Materials and Methods, Supplementary Figures, and Supplementary Table [file peerj-05-3732-s001.docx]

### Supplementary Materials and Methods

#### Taxonomic characterization of CCA communities

CCA communities were homogenized and DNA was isolated as described in Davies et al. (2013). The conserved 5′ portion of the eukaryotic small-subunit ribosomal RNA gene (18S SSU) was amplified via PCR following Davies et al. (2014), using primers (*SP-F-30* and *SP-R-540*) described in Vidal et al. (Vidal, Meneses & Smith, 2002). Successful amplification was detected for 6 out of the 8 assemblages from 2011, and for 4 out of the 6 CCA assemblages from 2012 (Supplementary Table S1). Amplicons were cleaned using PCR clean-up kits (Fermentas), and 10 ng of product served as template in the subsequent PCR to incorporate 454-rapid adapters and sample-specific barcodes. Each PCR contained 0.1 µM of the universal *Btn-SPR-F* forward primer (5′ CCT ATC CCC TGT GTG CCT TGG CAG TCT CAG TCT CAA AGA CTA AGC CAT GC 3′; underlined section matches *SP-F-30* primer) and 0.1 µM of unique reverse primer, which incorporated a unique 8-bp barcode for each CCA sample (5′ CCA TCT CAT CCC TGC GTG TCT CCG ACG ACT **AGAGACTC** TT ACA GAG CTG GAA TTA CCG 3′; underlined section matches *SP-R-540* primer; bold indicates a sample barcode) (Supplementary Table S1). Barcoding cycling profiles were 95°C for 5 min, four cycles of (95°C for 30 s, 55°C for 30 s, 72°C for 60 s) and 72°C 5 min. Resulting amplicons were gel-purified and sequenced using 454-FLX (Roche) with Rapid chemistry at the Genome Sequencing and Analysis Facility (GSAF) at the University of Texas at Austin. All raw sequence files are available in the National Center for Biotechnology Information’s BioProject archive under accession number PRJNA331102 (Supplementary Table S1).

### Supplementary Figures


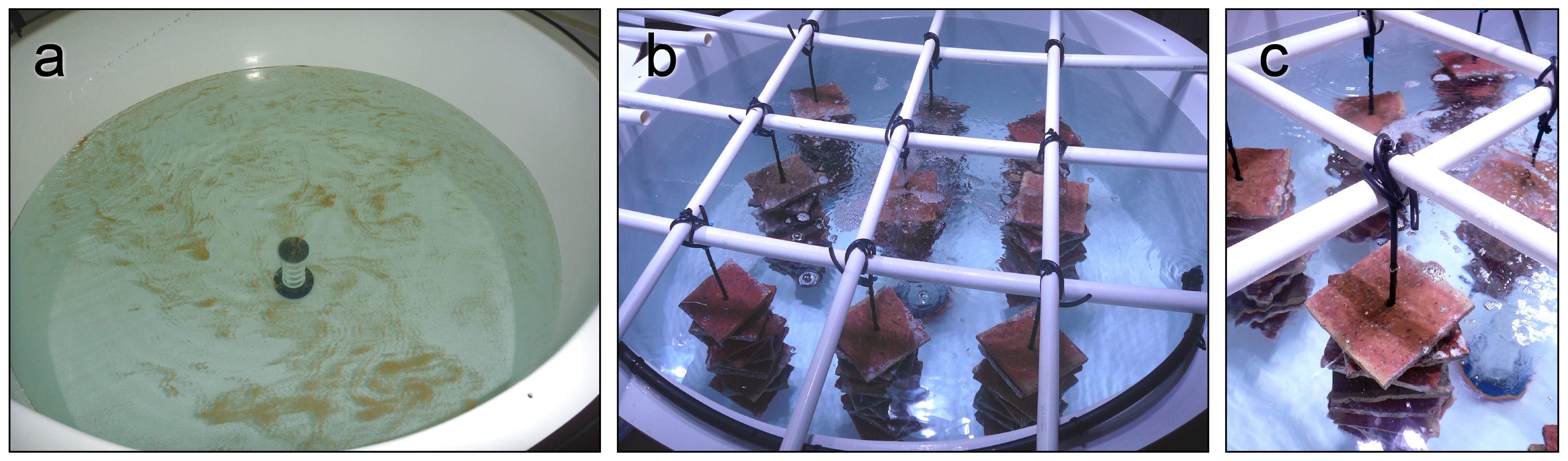


**Supplementary Figure S1**. Photographs of larval culture tanks showing a) central drain connected to an external standpipe to control water level within the tank that is covered with a plankton mesh filter to prevent loss of embyros and larvae. b,c) Terracotta settlement tiles are suspended in culture tanks with 3 cm long plastic spacers separating consecutive tiles.


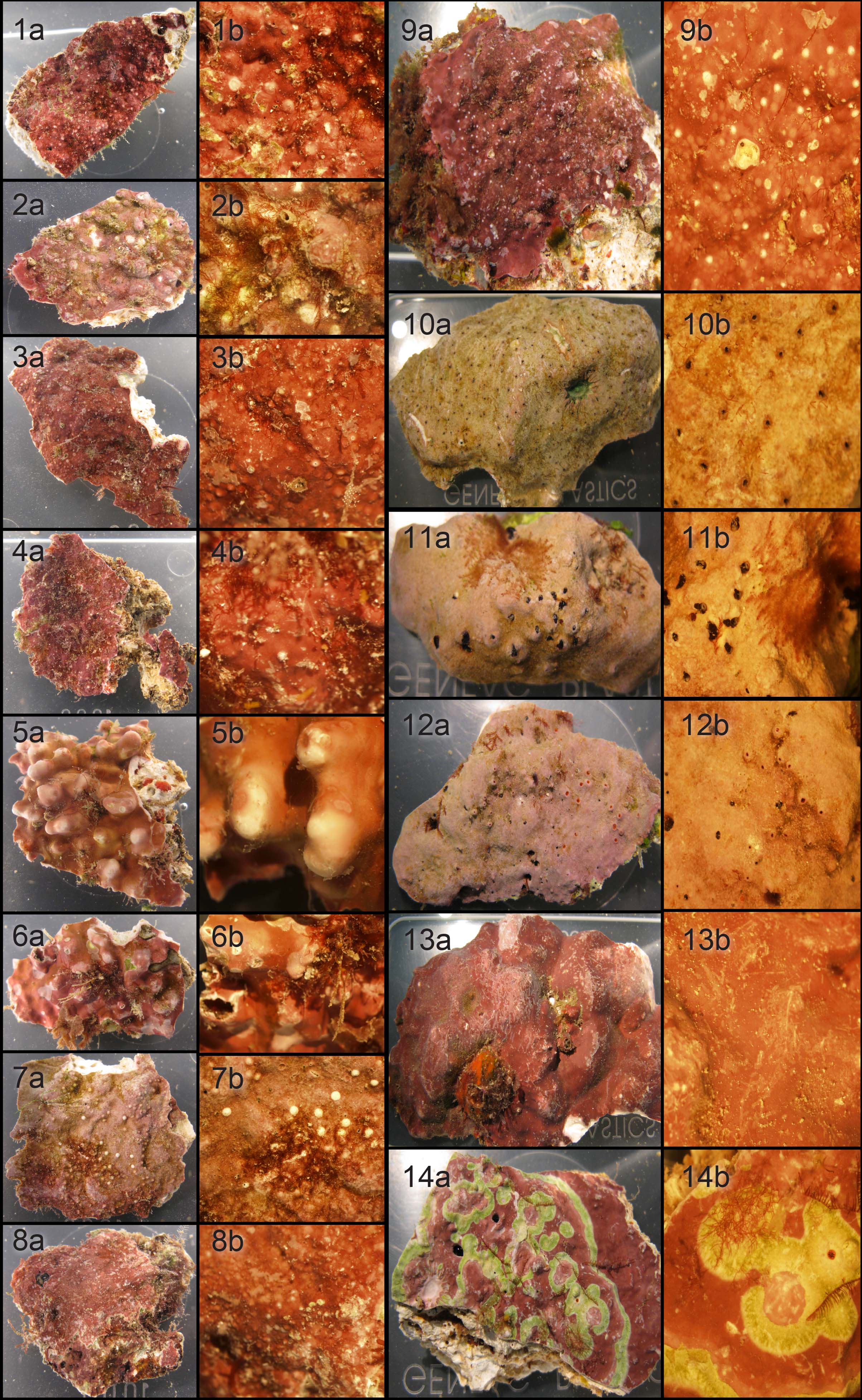


**Supplementary Figure S2**. Photographs of crustose coralline algae tested in 2011 (fragments 1-8) and 2012 (fragments 9-14), showing overall (a) and detailed (b) morphologies.

### Supplementary Tables

**Supplementary Table S1**: Summary of the metabarcoding libraries for the conserved 5′ portion of the eukaryotic small-subunit ribosomal RNA gene (18S SSU), including: sample name, barcode used for sequencing, BioSample Accession number, cycle number used for amplification, total number of raw reads, and total number of reads mapping to the top two clusters identified.

| **CCA sample** | **Barcode** | **BioSample accession** | **Cycle #** | **Raw reads** | **Reads mapped to top 2 clusters** |
| --- | --- | --- | --- | --- | --- |
| 2011_1 | AGAGACTC | SAMN05163306 | 24 | 2390 | 80.1 |
| 2011_2 | AGCTCGTG | SAMN05163307 | 21 | 5895 | 82.9 |
| 2011_3 | AGTGTCGA | SAMN05163308 | 17 | 2677 | 89.4 |
| 2011_4 | GAGCGCGC | SAMN05163309 | 27 | 2720 | 41.4 |
| 2011_5 | NA | NA | NA | NA | NA |
| 2011_6 | NA | NA | NA | NA | NA |
| 2011_7 | GATGAGTG | SAMN05163310 | 34 | 5233 | 68.7 |
| 2011_8 | GCGAGAGA | SAMN05163311 | 21 | 2259 | 83.5 |
| 2012_9 | NA | NA | NA | NA | NA |
| 2012_10 | AGAGACTC | SAMN05163312 | 29 | 966 | 88.0 |
| 2012_11 | AGCTCGTG | SAMN05163313 | 19 | 279 | 75.6 |
| 2012_12 | AGTGTCGA | SAMN05163314 | 21 | 418 | 79.2 |
| 2012_13 | NA | NA | NA | NA | NA |
| 2012_14 | GATGAGTG | SAMN05163315 | 29 | 603 | 90.5 |
